# Preparation of Porous Silicate Cement Membranes via a One-Step Water-Based Hot–Dry Casting Method

**DOI:** 10.3390/membranes12090838

**Published:** 2022-08-28

**Authors:** Zhantong Sun, Xiaojuan Wang, Haifeng Yuan, Shizhong Sang, Huacheng Xu, Yijun Huang, Congjie Gao, Xueli Gao

**Affiliations:** 1 Frontiers Science Center for Deep Ocean Multispheres and Earth System, Key Laboratory of Marine Chemistry Theory and Technology, Ministry of Education, College of Chemistry and Chemical Engineering, Ocean University of China, Qingdao 266100, China; 2 SEPCOIII Electric Power Construction Co., Ltd., Qingdao 266100, China; 3 Quanzhou Lanshen Environmental Protection Research Institute Co., Ltd., Quanzhou 362000, China

**Keywords:** hot–dry casting, cementitious materials, cement hydration, membrane performance

## Abstract

A commercial interest in the improvement in the separation performance and permeability of porous materials is driving efforts to deeply explore new preparation methods. In this study, the porous silicate cement membranes (PSCMs) were successfully prepared through an adjustable combination of hot–dry casting and a cement hydration process. The obtained membrane channel was unidirectional, and the surface layer was dense. The physical characteristics of the PSCMs including their pore morphology, porosity, and compressive strength, were diversified by adjusting the solid content and hot–dry temperature. The results indicated that with the solid content increasing from 40 wt. % to 60 wt. %, the porosity decreased by 8.07%, while the compressive strength improved by 12.46%. As the hot–dry temperature increased from 40 °C to 100 °C, the porosity improved by 23.04% and the BET specific surface area and total pore volume enlarged significantly, while the compressive strength decreased by 27.03%. The pore size distribution of the PSCMs exhibited a layered structure of macropores and mesopores, and the pore size increased with the hot–dry temperature. Overall, the PSCMs, which had typical structures and adjustable physical characteristics, exhibited excellent permeability and separation performance.

## 1. Introduction

Advantageous features including a smaller occupied area, lower energy consumption, and higher separation efficiency have made membrane separation technologies crucially important in recent decades for applications in various areas, such as gas separation, sewage treatment, oil–water separation, and seawater desalination [1,2,3]. Although membrane separation technology based on organic membranes has achieved much [4,5,6,7,8,9], organic membranes are known to be plagued with some inherent limitations, such as a poor resistance to alkali and an inability to bear high temperatures.

Due to these limitations of organic membranes, inorganic membranes have gained much popularity, particularly ceramic membranes which possess the beneficial characteristics of organic solvent resistance and outstanding thermal and chemical properties [10,11,12,13,14,15,16]. Currently, ceramic membranes primarily consist of typical oxides (silica, alumina, titania, and zirconia) and admixtures of these compounds [17,18,19,20]. Due to their excellent stability and favorable fouling resistance, ceramic membranes have become good candidates for water treatment, biological fermentation, gas separation, etc. [21,22,23]. However, both extremely high sintering temperatures and high-cost raw materials are typically required to fabricate these porous ceramic membranes, which involves multiple, complex steps [24,25]. Moreover, the resulting high fabrication cost and the limited types of membrane materials immensely restrict their application. Therefore, the global demand for state-of-the-art membrane materials that have both excellent properties and ultralow costs continues to rise sharply and represents a momentous challenge facing the world.

Silicate cement, as an indispensable and conventional engineering material produced in vast quantities, has played a vital role in exploring novel types of materials to prepare membranes [26,27,28]. Cementitious materials are extremely complex composites, which exhibit the characteristics of their different components, interfaces, and microstructures. Moreover, since cement is easy to shape, setting with time, hardening, and gradually increasing in strength when mixed with water, research on fabricating cement-based porous separation membranes is remarkably valuable for making use of their low-energy consumption, operational simplicity, and small occupied area [1,29]. The greatest advantage of porous silicate cement membranes is that they can be modified by adding any other materials, in contrast with ceramic membranes. The emergence of novel membranes with cementitious materials broadens their scope of application and makes up for the limitations of existing ceramic membranes.

In our previous studies [30,31,32], porous silicate cement compacts (PSCCs) were successfully prepared by means of a freeze-casting technique. To be specific, PSCCs with diversified pore sizes were fabricated via the adjustment of solid content, freezing temperature, and the type of pore-forming agent. Nevertheless, there was still room for improvement in such aspects as the relatively complex process and long preparation time. Additionally, casting techniques related to preparing cement membranes are so scarce that there is an urgent need for their exploration. Considering the cement curing process in the traditional construction field, hot–dry curing makes cement slurries lose part of their water and form capillary pores, which significantly affects the hydration reaction and cement structure. This is unfavorable for improving the mechanical properties of cement and therefore the demand for high strength in the construction field cannot be met. However, hot–dry curing can greatly shorten the curing period and forms a certain pore structure that we need. With this method, only one oven is required to successfully prepare the cement membrane, which greatly saves on equipment costs.

Hence, in this paper, hot–dry casting is first proposed as a method to fabricate porous silicate cement membranes (PSCMs) with favorable permeability and regular-shaped pore channels. The effects of solid content and hot–dry temperatures on the PSCM physical characteristics were investigated. Techniques including scanning electron microscopy (SEM), X-ray diffraction (XRD), and mercury intrusion porosimetry (MIP) were employed to characterize the microstructure of the PSCMs.

## 2. Materials and Methods

### 2.1. Materials

Commercial silicate cement (P.O 52.5, Sunnsy group, Qingdao, China) was used as the membrane-forming raw materials in this study. Deionized water (DI, 18.2 MΩ•cm) was used as the pore-forming agent. Bovine serum albumin (BSA, 67000 Da; Sinopharm Chemical Reagent Co., Ltd., Shanghai, China) was used as the test solution. The primary chemical components of the silicate cement powder used in this paper are specified in Table 1.

### 2.2. Preparation of PSCMs

**Figure 1:**
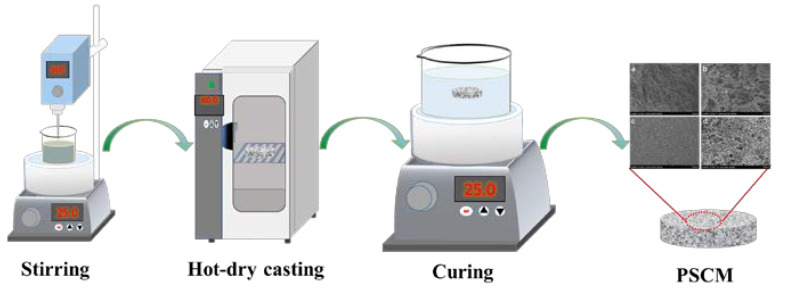
Schematic diagram of the hot–dry casting technique for fabricating the porous silicate cement membrane (PSCM).

The commercial silicate cement powder (P.O 52.5) was ground with a ball mill for 12 h to make the cement powder more uniform and compact. Then, the ball-milled silicate cement powder and deionized water were mixed evenly in a certain proportion of 40 wt. %, 50 wt. %, 60 wt. %, stirred, and degassed in a vacuum. The mixed silicate cement paste was poured into a self-made Teflon mold and then sealed into the vacuum drying oven with a temperature of 40 °C, 60 °C, 80 °C, and 100 °C for 10 h each. After the curing process (humidity: 100%, temperature: 25 °C), which lasted 20 days to ensure the complete hydration reaction, the PSCM was acquired. A schematic diagram of the hot–dry casting technique for fabricating the PSCM is shown in Figure 1.

### 2.3. Characterizations of PSCMs

The phase identification of the PSCMs was carried out using X-ray diffraction (XRD, D8 Advance, Bruker AXS GmbH, Karlsruhe, Germany) with a scan range from 5° to 80°. The chemical structure of the PSCMs was analyzed via Fourier transform infrared spectroscopy (FTIR, Tensor 27, Bruker Optik GmbH, Ettlingen, Germany). After cracking and smoothing, surface and cross-sectional morphologies of the PSCMs were obtained with scanning electron microscopy (SEM, HITACHI S-4800, Hitachi, Tokyo, Japan). Nitrogen adsorption/desorption isotherm of the PSCMs was detected with an automated surface and pore size analyzer (Quantachrome, NOVA 2200e, Boynton Beach, FL, USA). The density functional theory (DFT) was employed to calculate the pore width, and the specific surface area of the PSCMs was determined by the Brunauer, Emmett, and Teller (BET) method. Mercury intrusion porosimetry (MIP, AutoPore IV 9500, Norcross, GA, USA) was carried out to analyze the pore size distribution of the PSCMs. The porosity of the PSCMs was determined by the Archimedean principle. The compressive strength of the PSCMs cured to the set age was measured using the compression-testing machine (TYE-300, JIANYI Experiment Instrument Co., Ltd, Wuxi, China). The average compressive strength and standard deviations were determined by testing five samples under the same conditions.

### 2.4. Membrane Performance Measurements

The permeation performance of the PSCM was determined by pure water flux and BSA solution flux via the cross-flow membrane filtration apparatus under a set value at ambient temperature. In brief, the PSCM was primarily compacted at 0.15 MPa for 30 min. Afterwards, both the pure water flux and BSA solution flux of the PSCM were tested at 0.1 MPa and calculated according to the following Equation (1) [9]:

(1) $$J=\frac{m}{\rho \cdot A\cdot t}$$

where J is the pure water flux or BSA solution flux of the PSCM, L·m^−2^·h^−1^; *m* is the weight of permeate water, kg; *ρ* is the density of pure water at 25 °C, kg·L^−1^; *A* is the effective filtration area, m^2^; and *t* is the penetration time, h.

The BSA rejection ratio (*R*), one of the most direct indicators of membrane performance, was determined by equation (2) [33]:

(2) $$R=\left(1-\frac{C_{p}}{C_{f}}\right)\times 100\%$$

where $C_{p}$ and $C_{f}$ are the BSA concentrations in the permeate and feed solution, respectively, which were measured with an ultraviolet visible spectrophotometer (UV-2450, Shimadzu Co., Ltd., Kyoto, Japan).

## 3. Results and Discussion

### 3.1. Chemical Structure Analysis

**Figure 2:**
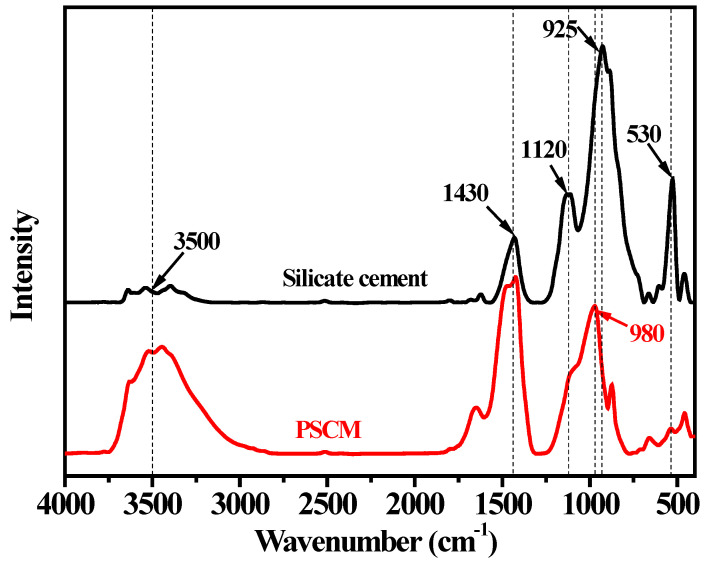
FT-IR spectra of silicate cement and PSCM based on the hot–dry casting technique.

The silicate cement mainly consisted of massive Alite (925 cm^−1^, 530 cm^−1^) and a certain proportion of carbonate (1430 cm^−1^) and gypsum (1120 cm^−1^). The difference between silicate cement and PSCM was investigated by FT-IR, as shown in Figure 2. The spectral band changed clearly with the hydration process. The characteristic peak of Alite (530 cm^−1^) corresponded to the surface of the curved vibration of the silicon tetrahedron, and the relative strength of this band rapidly reduced after the hydration process. The peak of the band moved from 925 cm^−1^ to 980 cm^−1^, which corresponded to the characteristic band of C-S-H. This change indicated that the hydration process could be used to generate the hydration products, C-S-H. Additionally, 3500 cm^−1^ represented a hydroxyl vibratory band of the hydration product, which was the characteristic mark of the hydration reaction.

### 3.2. Phase Analysis

**Figure 3:**
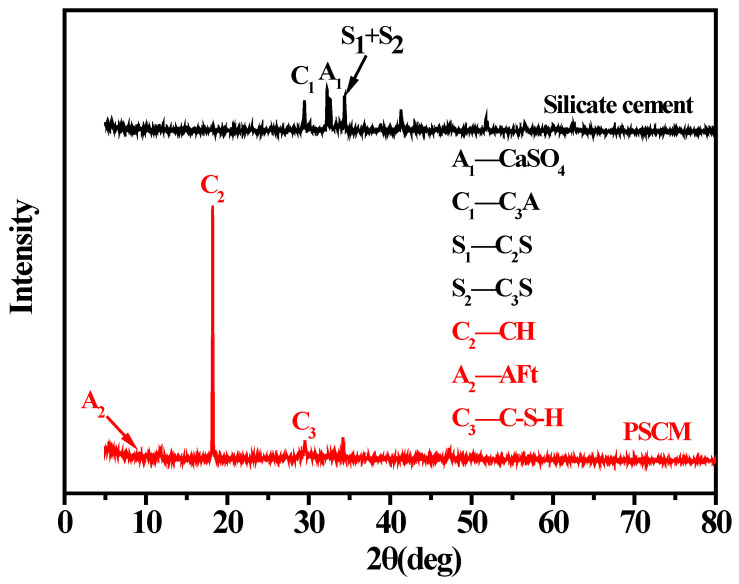
XRD of the silicate cement and PSCM based on the hot–dry casting technique.

In this study, XRD was employed to determine the similarities and differences between the silicate cement and the PSCM prepared with 50 wt. % solid content. As one kind of high-performance hydraulic cementitious material, silicate cement contains many minerals, such as tricalcium silicate (C_3_S), dicalcium silicate (C_2_S), and tricalcium aluminate (C_3_A), etc. It can be seen from Figure 3 that these components were transformed into a large amount of hydration products—including calcium hydroxide (CH), ettringite (AFt), and calcium-silicate-hydrate (C-S-H)—after 20 days of hydration reaction. This was attributed to the hydration reaction in fabricating the PSCM, which made the corresponding mineral phase diffraction peak weaken or disappear. Furthermore, the phase diffraction peak of the PSCM exhibited favorable agreement with the silicate cement compacts under standard conditions [30]. This demonstrated that the hot–dry casting technique had almost no impact on the hydration reaction of silicate cement, and the resulting primary hydration products remained unchanged. Using different casting parameters, including the solid content and hot–dry temperature, similar XRD spectra could be acquired in this experiment.

### 3.3. Micro-Morphological Characteristics

**Figure 4:**
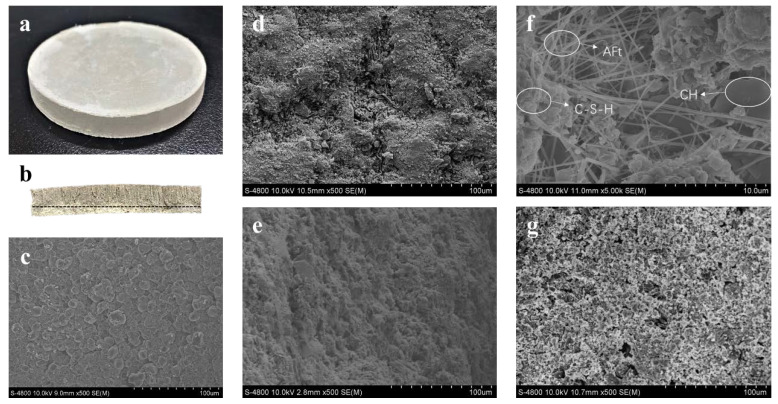
SEM images of the PSCM under the conditions of 50 wt. % and temperature of 100 °C based on the hot–dry casting process: (**a**) the overall morphological structure, (**b**) the cross section, (**c**) the bottom surface, (**d**) the cross-section of the separation layer, (**e**) the vertical cross section and (**f**) its details, and (**g**) the upper surface.

The micro-morphological characteristics of the PSCM under the conditions of 50 wt. % solid content and a temperature of 100 °C, based on the hot–dry casting process, are illustrated in Figure 4. Figure 4a shows the overall morphological structure of the PSCM, and Figure 4b exhibits its cross-section with an obvious layered structure. Figure 4c shows the smooth bottom surface of the PSCM; its shape was a dense layer with uniform pore morphology and fewer pores, which was considered an excellent separation functional layer. Figure 4d displays the cross-section of the PSCM separation layer with compact and uniform pores, laying the foundation for a good separation performance. It was evident that the PSCM showed directional porosity (Figure 4e), which was consistent with the track of water evaporation. In the hot–dry casting process, water in the cement evaporated from bottom to top under the action of thermal traction to form a certain pore structure, while particles in the slurry were repelled and became more compact. This is mainly because of unceasing dehydration during cement hydration in the process of hot–dry casting. It is well known that cement pastes should be in a state of full moisture during hot–dry casting and that porous structures form because of early dehydration. After the free water among cement particles evaporated, capillary pores formed which could not be filled by hydration products at the later stage. This is owing to the fact that the transfer of hydration products can only be carried out by solid dissolution in liquid phase and then nucleation and crystallization in supersaturated solution. Figure 4f is the detailed image of Figure 4e. It shows that the inner PSCM possessed a wealth of hydration products, such as short rod-shaped AFt, hexagonal lamellar CH, and a three-dimensional network of C-S-H gel [33,34]. Unlike the bottom surface, there are some randomly arranged pores on the upper surface of the PSCM (Figure 4g). This was due to water vapor evaporating from the bottom up and irregular pores generating when the superstratum water vapor escaped from the upper surface. Certainly, these larger pores contributed to improving the permeability of the PSCMs.

### 3.4. Affect of Solid Content on the PSCM

**Figure 5:**
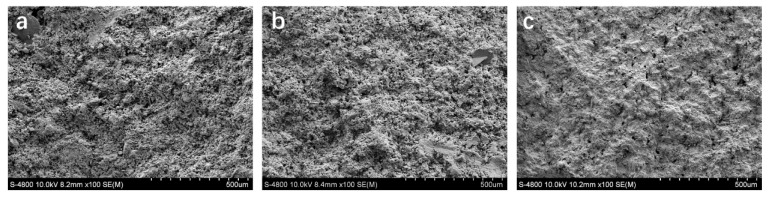
SEM images of the PSCM with the hot–dry temperature of 40 °C and the solid content of (**a**) 40 wt. %, (**b**) 50 wt. %, and (**c**) 60 wt. %, based on the hot–dry casting process.

In the hot–dry casting process, DI water as a pore-forming agent was the main factor affecting the structure and performance of the membrane. In order to investigate the effect of different solid contents on the pore channel of the PSCM, the hot–dry temperature was fixed at 40 °C. Then, water with different volume fractions was added to the premixed cement slurry as the pore-forming agent to prepare the PSCM. Figure 5 shows the SEM images of the PSCM based on the hot–dry casting process at different solid contents of 40 wt. %, 50 wt. %, and 60 wt. %. It is obvious that the PSCM presented a coral-like pore structure, and, with the increase in solid content, the PSCM became denser. The higher the solid content, the greater was the resistance to water evaporation, and the less was the possibility of pore formation. Conversely, when the solid content was low, that is, when cement particles were packed loosely, the moisture among the cement particles evaporated and formed the capillary pores as the temperature increased.

**Figure 6:**
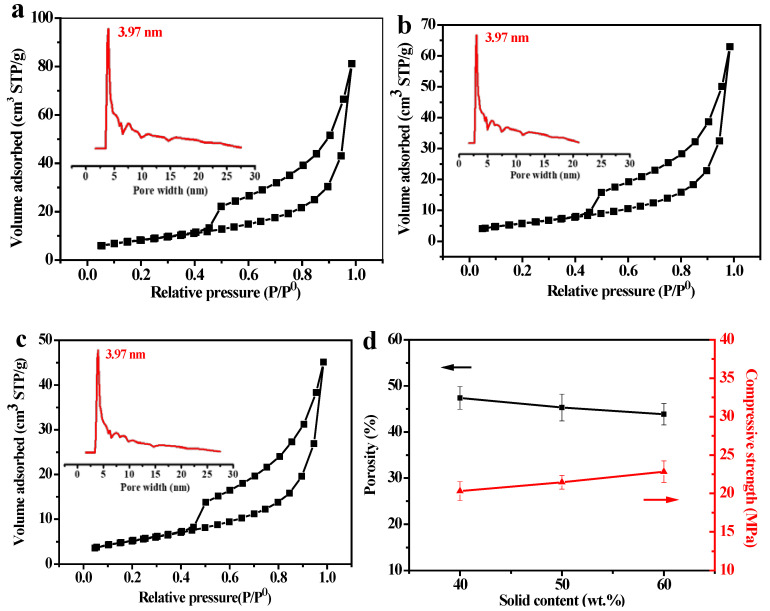
Nitrogen adsorption–desorption curve (in black) and mesoporous pore size distribution (in red) of the PSCM based on the hot–dry casting process with solid contents of (**a**) 40 wt. %, (**b**) 50 wt. %, and (**c**) 60 wt. %. (**d**) Porosity and compressive strength at different solid contents.

The mesoporous distribution, specific surface area, and pore volume of PSCMs with different solid contents were measured by the nitrogen adsorption method, as displayed in Figure 6a-c. The results showed that the PSCM possessed a classic type II nitrogen adsorption–desorption isotherm, and its H3 hysteresis loop indicated that there was a well-developed mesoporous structure in the PSCM. Despite the variations in solid content, the PSCMs exhibited a consistent pore size of 3.97 nm, which displayed a limited and weak relationship between variations in solid content and the mesoporous size of the PSCM. As the solid content increased from 40 wt. % to 60 wt. %, the specific surface area of the PSCM decreased from 30.06 m^2^·g^−1^ to 19.03 m^2^·g^−1^, while the total pore volume decreased from 0.10 cm^3^·g^−1^ to 0.06 cm^3^·g^−1^. As mentioned above, this result is strongly related to the increase in the density of the PSCM due to the increase in the solid content [35]. Moreover, the variations in solid content can also regulate the compressive strength and porosity of the PSCM. As shown in Figure 6d, with the increase in solid content the porosity of the PSCM decreased from 47.38% to 43.84%, while the compressive strength increased from 20.31 MPa to 22.84 MPa. Thus, the increase in solid content can improve the compactness of the PSCM, which provides better mechanical properties. As a pore-forming agent, water is beneficial for permeation performance by virtue of its molecular morphology. The higher the water content, the larger the pore volume and porosity, which in turn reduces the mechanical strength of PSCMs. Hence, there is a trade-off between high porosity and high mechanical strength.

### 3.5. Affect of the Hot–Dry Temperature on the PSCM

**Figure 7:**
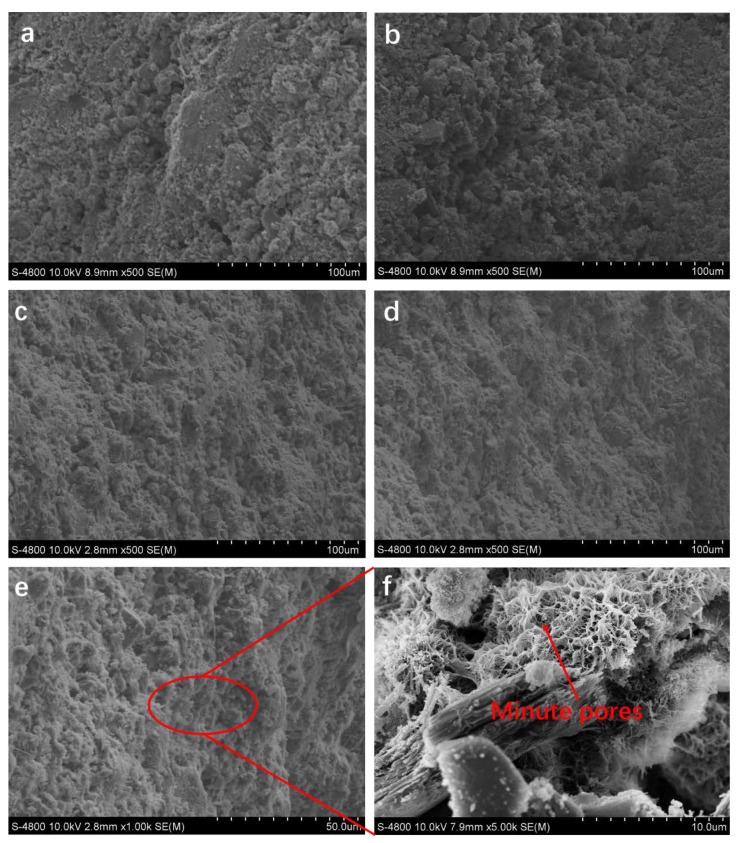
SEM images of the PSCM based on the hot–dry casting process at (**a**) 40 °C, (**b**) 60 °C, (**c**) 80 °C, and (**d**) 100 °C. (**e**) The surface of the pore structure and (**f**) its detail.

In the process of membrane preparation, the parameter of hot–dry temperature exhibited a direct influence on the morphology of the pore channel, thereby affecting the structure and performance of the membranes. With the solid content at 50 wt. %, experiments and characterizations were carried out to further explore the influence of different hot–dry temperatures on the preparation of PSCM. SEM images of the cross-sections of PSCMs at 40 °C, 60 °C, 80 °C, and 100 °C based on the hot–dry casting process are shown in Figure 7. It can be seen that there were certain pore channels in all the samples prepared under these temperatures, and with the increase in hot–dry temperature, the directional channels became more obvious.

At a relatively low temperature, the temperature gradient and heat transfer efficiency were low, leading to a relatively slow water evaporation rate, which made it easier to form a larger oval foramina structure (Figure 7a,b). As shown in Figure 7c,d, when the temperature was relatively high, the directional growth temperature gradient was large, and free water evaporated at a rapid rate, thus forming an interconnected coral-like pore structure. Moreover, the surface of the pore structure displays a particular concave and convex morphology with abundant pore channels (Figure 7e). Figure 7f is a higher magnification of the pore structure, which was composed of many minute pores.

**Figure 8:**
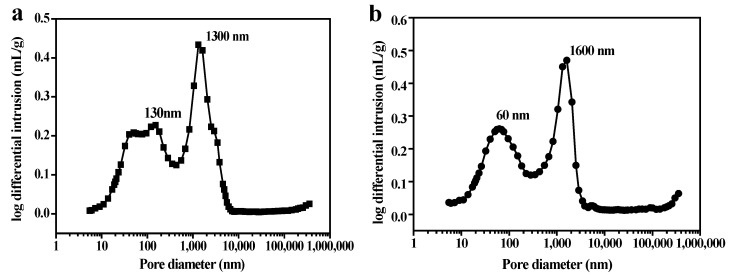

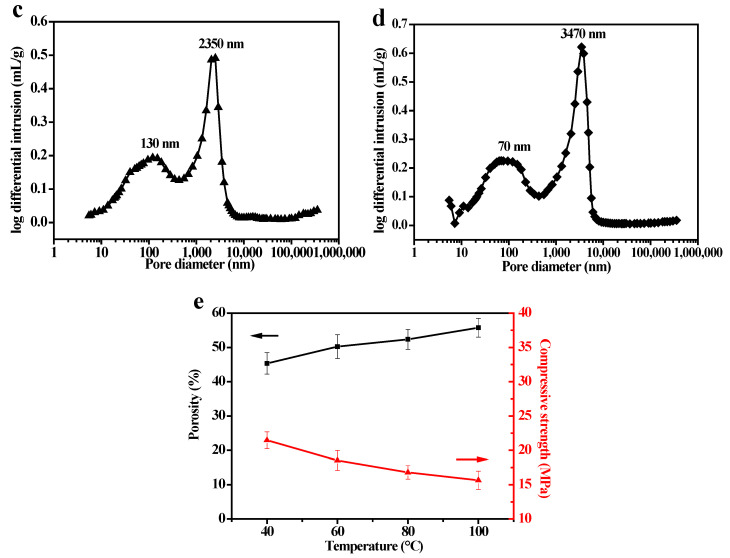
Pore size distribution of PSCMs based on the hot–dry casting process at (**a**) 40 °C, (**b**) 60 °C, (**c**) 80 °C, and (**d**) 100 °C. (**e**) Porosity and compressive strength at the above hot–dry temperatures.

Mercury intrusion porosimetry was employed to further illustrate the pore size distributions of PSCMs at different temperatures (40 °C, 60 °C, 80 °C, 100 °C). As shown in Figure 8a–d, there was typical bimodal pore size distribution in the PSCMs. The highest peak values of the PSCMs prepared at different temperatures were 1300 nm (40 °C), 1600 nm (60 °C), 2350 nm (80 °C), and 3470 nm (100 °C), indicating that the pore size of the PSCMs could be improved with an increase in temperature even if the secondary modification of the pore was made by silicate cement hydration products. The porosity and compressive strength in the PSCMs at different temperatures are illustrated in Figure 8e. Through analysis, it was found that the porosity of the PSCM increased from 45.32% to 55.76% with the increase in temperature, while the compressive strength decreased from 21.46 MPa to 15.66 MPa. Owing to the small traction force at low temperatures, water did not easily evaporate from the cracks of the cement particles, leading to a denser structure or higher compressive strength [36]. However, when the temperature was high, water vapor volatilized into the cement particle gaps and many pores remained under great traction. Moreover, the accumulation of the cement particles was loose, which resulted in the low compressive strength of the PSCM [37].

**Figure 9:**
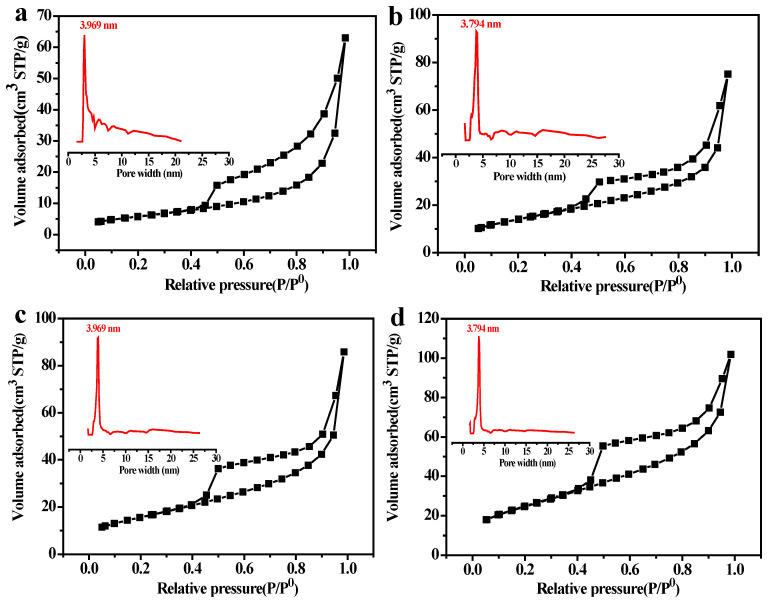
Nitrogen adsorption–desorption curve (in black) and mesoporous pore size distribution (in red) of the PSCMs based on the hot–dry casting process at (**a**) 40 °C, (**b**) 60 °C, (**c**) 80 °C, and (**d**) 100 °C.

In this experiment, the mesoporous distribution, specific surface area, and pore volume of the PSCMs under different temperatures were tested by the nitrogen adsorption method. It can be seen from Figure 9 that the PSCMs exhibited the classic type II nitrogen adsorption–desorption isotherm, and the H3 hysteresis loop indicated that the PSCMs possessed a well-developed mesoporous structure. All these PSCMs showed a similar pore size distribution of roughly 4 nm at different hot–dry temperatures.

In the casting process of the PSCMs, the secondary modification of the pore walls by hydration products and the formation of capillary pores after the evaporation of water among cement particles can lead to the formation of mesoporous pores. With the temperature increasing from 40 °C to 100 °C, the specific surface area of the PSCMs increased from 21.13 m^2^·g^−1^ to 89.65 m^2^·g^−1^, while the total pore volume increased from 0.07 cm^3^·g^−1^ to 0.13 cm^3^·g^−1^. This result showed a strong relationship between the decrease in the density of the PSCMs and the increase in hot–dry temperature.

### 3.6. Performance Test of the PSCMs

**Figure 10:**
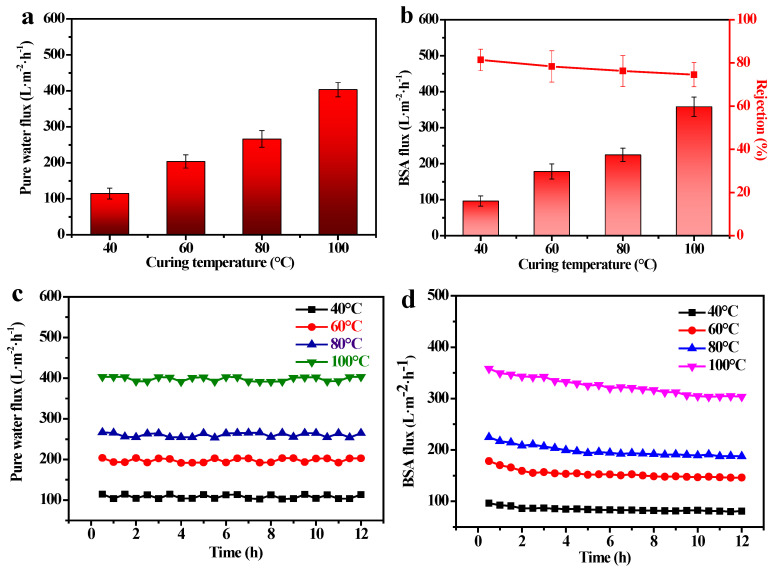
Permeability and separation performance of PSCMs based on the hot-dry casting at different temperatures: (**a**) the pure water flux of the PSCMs, (**b**) the BSA flux and rejection rate of BSA solution, (**c**) the pure water flux stability test of the PSCMs, and (**d**) the BSA flux stability test of the PSCMs.

In this experiment, PSCMs with a solid content of 50 wt. % and temperatures of 40 °C, 60 °C, 80 °C, and 100 °C were selected for an evaluation of their membrane performance. It was found that the pure water flux of the PSCMs increased from 114.65 L·m^−2^·h^−1^ to 403.40 L·m^−2^·h^−1^ with the increase in temperature (Figure 10a). This was because an increase in temperature accelerated water evaporation inside the PSCMs, whereby water among the cement particles evaporated to form capillary pores. The higher the temperature, the greater the traction on water and the more pores formed, leading to the increase in the pore size and permeability of the PSCM. Figure 10b shows the flux and rejection rate of BSA solution (0.1 g·L^−1^) at different temperatures under the above test conditions. The results showed that the BSA flux of the PSCM increased with the temperature, while the rejection rate decreased slightly. At the temperature of 40 °C, the maximum BSA rejection rate of the PSCM based on the hot–dry casting process was 81.42%, and the corresponding BSA flux was 96.37 L·m^−2^·h^−1^. This was because, under the low temperature, water with low traction can hardly evaporate from the cement particles to form capillary channels, which improved the rejection ability of the PSCM but inevitably led to the increase in mass transfer resistance. Compared with other well-known ceramic membranes [38], the PSCMs showed lower flux yet better retention performance. More research should be conducted to further enhance the water flux in subsequent experiments. The flux stability test of the PSCMs based on the hot–dry casting process was demonstrated in Figure 10c,d. It can be seen that PSCMs prepared under different hot–dry temperatures exhibited favorable pure water flux stability in Figure 10c. However, there was a large decline in the BSA flux stability, as shown in Figure 10d, owing to the membrane fouling.

## 4. Conclusions

In this paper, PSCMs with regular-shaped pore channels was successfully prepared via the hot–dry casting technique, which exhibited the characteristics of high cost efficiency, simple casting, and excellent performance. The "designed" membrane pore structure was obtained by combining the pore channels left by water evaporation with the final cement hydration reaction. The physical properties of the PSCMs such as pore size, pore morphology, and porosity, could be effectively controlled by adjusting the solid content in the cement reaction system and the temperature of the hot–dry casting process. Classic type II nitrogen adsorption–desorption isotherm was shown in the PSCMs, and its H3 hysteresis loop indicated that there was a well-developed mesoporous structure. With the increase in hot–dry temperature and the decrease in solid content, the specific surface area exhibited a corresponding increase. Most significantly, the PSCMs displayed outstanding permeability and separation performance. With the continuous increase in hot–dry temperature, the pure water flux of the PSCMs increased sharply from 114.65 L·m^−2^·h^−1^ to 403.40 L·m^−2^·h^−1^. The maximum BSA rejection rate of the PSCMs reached 81.42% and its corresponding BSA flux was 96.37 L·m^−2^·h^−1^. Moreover, PSCMs prepared under different hot–dry temperatures exhibited favorable flux stability. In addition, this casting technique was less time-consuming, thus saving more than one-third in time cost in contrast with freeze casting because it can be accomplished in only one step. These findings propose a new avenue to fabricate cement-based membranes featuring more modification possibilities, such as combining nanomaterials with the initial cement slurries or directly impregnating them on the surface of PSCMs.

## Figures and Tables

**Table 1:** The primary chemical components of silicate cement powder.

Components	CaO	SiO_2_	Fe_2_O_3_	Al_2_O_3_	MgO
Content (wt. %)	62.47	22.15	5.73	4.03	3.37

## Data Availability

Not applicable.
